# Role of the Surface in Conformational Changes in Lysozymes: Effect of a Gold Surface and a Lipid Membrane

**DOI:** 10.3390/ijms262311303

**Published:** 2025-11-22

**Authors:** Agnieszka Kaminska, Lukasz Lustyk, Jacek Gurgul, Barbara Jachimska

**Affiliations:** Jerzy Haber Institute of Catalysis and Surface Chemistry Polish Academy of Sciences, Niezapominajek 8, 30-239 Krakow, Polandjacek.gurgul@ikifp.edu.pl (J.G.)

**Keywords:** lysozyme, adsorption, lipid membrane, gold surface

## Abstract

The study of the conformational stability of protein layers at the interface between gold surfaces and lipid membranes is crucial for determining the biological activity of these systems and understanding their interactions. The surfaces differ significantly in hardness: gold is a rigid substrate, while the POPC/POPS liposome layer is highly flexible. A quartz crystal microbalance with dissipation (QCM-D) monitoring method and multi-parametric surface plasmon resonance (MP-SPR) were used to determine the adsorption efficiency of lysozymes, the level of layer hydration, and changes occurring within the secondary structure and the thickness of the formed protein layer. In both methods, lysozyme adsorption on the gold surface was more effective at pH 4.0 than at pH 7.4. The lysozyme adsorption efficiency on the surface of the lipid layer was the same for both measurement conditions. In contrast, the affinity of lysozyme molecules to the lipid surface was higher than that of the gold surface. The composition of the secondary structure of lysozymes was monitored using the FT-IR method. Deconvolution of the Amide I band confirms the existence of different mechanisms underlying lysozyme molecule immobilization depending on the type of adsorption surface. Along with the change in the surface, there is a transition from the dominance of electrostatic to hydrophobic interactions, which significantly affects the structure of the interphase layer. High content of random structures on the lipid surface is evident, while, in the case of the gold surface, there is a decrease in random structures and the presence of antiparallel β-sheets. Interaction with the surface induces the transition of amyloidogenic domains of the protein to conformations, which are particularly susceptible to aggregation, consequently leading to oligomerization.

## 1. Introduction

The conformational stability of a protein is a crucial property, as it determines the protein’s functionality. Identifying the factors that trigger a cascade of changes leading to structural alterations in proteins, thereby affecting their biological function, is an essential research goal. Globular proteins are typically characterized by high interfacial activity, and their adsorption properties are complex due to the range of different conformations their molecules can adopt at interfaces. Although protein molecules can adsorb without significant changes in relation to their initial state, they may undergo various degrees of melting and unfolding of their structure, which can lead to the loss of biological functions, for example, blocking enzymatic activity due to the adoption of non-native conformations or obstruction of the active site. These misfolded states can involve a significant degree of intermolecular interactions, often leading to the formation of large numbers of intermolecular α-sheets. During the formation of intermolecular β-sheets, insoluble protein aggregates are formed, which are characteristic of amyloid and prion diseases. Protein–surface interactions play an essential role in various research areas, including biosensors, drug delivery systems, material functionalization, disease development, and many others [1,2,3]. The protein adsorption phenomenon has been extensively studied by numerous groups using advanced physicochemical methods, including ellipsometry, atomic force microscopy (AFM), surface plasmon resonance (SPR), optical waveguide light mode spectroscopy (OWLS), quartz crystal microbalance with dissipation (QCM-D) monitoring, and others [4,5,6].

Many factors influence the maintenance of protein conformational stability during the adsorption process, including protein properties (strength of intramolecular interactions within the protein, charge distribution, and concentration), solution conditions (ionic strength and pH), and surface properties (charge, roughness, and surface energy) [7,8]. Another critical factor affecting protein adsorption is the number of protein biomolecules, which translates into coverage of the surface and protein protonation, determining surface wettability. These factors influence the conformation of proteins—a crucial aspect in their toxicity, as secondary and tertiary structural changes can impact the development of cascading events that lead to disease in the human body [9,10,11]. Protein–surface interactions may lead to protein misfolding and aggregation, which are observed during the development of neurodegenerative diseases. Understanding the extent to which phenomena occurring at the interface can promote or inhibit protein misfolding allows us to determine the mechanisms underlying such processes.

It has also been demonstrated that protein–lipid interactions can induce structural changes and alter lipid bilayer properties, potentially leading to the development of diseases [12]. Lysozymes are small, alkaline proteins with a molecular weight of approximately 14.3 kDa, which are present in human physiological fluids such as tears, mucus, breast milk, or saliva. The most common commercially available lysozyme is obtained from hen egg white, but they can also be obtained from other birds’ eggs or even those of invertebrates [13]. These proteins are divided into two domains that are linked by a long α-helix, between which the active center is located in a deep cleft [14].

Lysozymes are widely used in medical diagnostics as a biomarker for many diseases (leukemia, Crohn’s disease, atherosclerosis, renal tubular damage, sarcoidosis, oral infections, and cancer) [15]. Au nanoparticles (AuNPs) are often used in lysozyme-based biosensors. Many research groups have studied the interactions between gold nanoparticles and lysozymes to understand the process of AuNP aggregation in the presence of proteins and the underlying mechanisms [16,17,18]. The use of gold nanoparticles in in vitro and in vivo systems is making it increasingly necessary to study the interactions between various biomolecules, especially those found in body fluids, with the aim of discovering potential new diagnostic methods. The research and analysis of specific biomolecules adsorbed to AuNPs allows for the identification of new molecular biomarkers for both diagnostic and therapeutic applications. The encapsulation of gold nanoclusters in protein systems (AuNCs) has significant application potential due to their unique fluorescent properties [19]. The presence and quantity of specific amino acid residues in proteins have been shown to play a significant role in the formation of gold nanoclusters. Nucleation of gold nanoclusters in the lysozyme structure is localized in the region of disulfide bridges. The formation of gold nanoclusters disrupts S–S bonds between cysteines, thereby destabilizing the protein structure, which, under these conditions, tends to form dimers/trimers or larger aggregates [20,21]. The effect of lysozyme susceptibility to amyloid formation is the occurrence of a disease known as “familial or systemic amyloidosis,” characterized by the formation of lysozyme amyloid deposits in the kidneys, liver, and spleen [22]. Understanding the fundamental mechanisms of protein misfolding and aggregation is crucial to elucidate the causes of neurodegenerative diseases. Literature data indicate that the formation of toxic oligomers—which are precursors to amyloid fibrils—is initiated at the cell membrane surface, rather than in the cytosol [23]. In particular, anionic phospholipids potentially increase the likelihood of amyloid fibril formation in proteins such as Aβ proteins, lysozymes, and α-synuclein [24].

Lysozymes are natural antibiotics with anti-inflammatory and antioxidant properties and are among the human body’s defense factors with nonspecific immunity mechanisms [25]. Moreover, the lysozyme dimer appears to modulate the synthesis of factors related to tumor necrosis, while lymphocytes stimulate the production of interleukins and interferons [13,26]. Lysozymes are among the proteins that are thought to participate in amyloidosis by preventing β-sheet formation in a binding pathway to monomeric Aβ species [27]. However, some studies suggest that although lysozymes can potentially protect other cells from amyloid fibrils, they can also create synthetic fibrils [27]. Due to their unique features, lysozymes can be studied as model proteins to investigate interactions with other biomolecules and interfaces [28].

Lipids and proteins are the main components of animal cell membranes [29]. The interaction of proteins with lipid membranes plays a significant role in various signaling pathways and cell–cell communication. Any change in the conformation of a protein, whether as an integral part of the cell membrane (e.g., a receptor) or as a signaling molecule, may lead to serious consequences for human health, such as cystic fibrosis or Stargardt disease [30,31]. Other studies suggest that protein interactions with the lipid interface may reveal the detailed pathogenesis mechanism of neurological disorders as lipid attachment to proteins enhances hydrophobicity, which is pivotal in regulating the stability, localization, signal transmission, and conformation of transmembrane proteins [32,33,34]. It can also help to clarify how the protein corona forms around nanoparticles [35]. Therefore, understanding how proteins interact is crucial for future development and the improvement of medical care. Some research groups have studied the interactions of proteins with lipids [36,37,38]; however, there is still a lack of comprehensive models for physicochemical characterization integrating different methods and approaches to fully elucidate the adsorption mechanism of proteins onto the lipid interface under various conditions.

The presented research provides essential information on the design of highly stable protein layers and the control of protein secondary structure in the context of neurodegenerative diseases or nanobiotechnology applications. The ability to modulate protein aggregation is crucial for discovering fundamental biological principles, elucidating disease mechanisms such as those involved in neurodegenerative diseases, and exploring therapeutic possibilities. By combining advanced physicochemical and spectroscopic characterization methods (UV–Vis, CD, DLS, zeta potential measurement, QCM-D monitoring, MP-SPR, FT-IR, and XPS), it was possible to control the conformational stability of lysozymes at the phase boundary under various adsorption conditions. The study of protein systems in microfluidic systems, such as those used in the QCM-D and SPR methods, is of great importance as it enables the analysis of systems under dynamic flow conditions, which is crucial from a biological point of view. Moreover, the unique properties of adsorbed protein layers may enable the development of functional biomaterials.

## 2. Results and Discussion

### 2.1. Physicochemical Characteristics of Lysozymes

**Figure 1:**
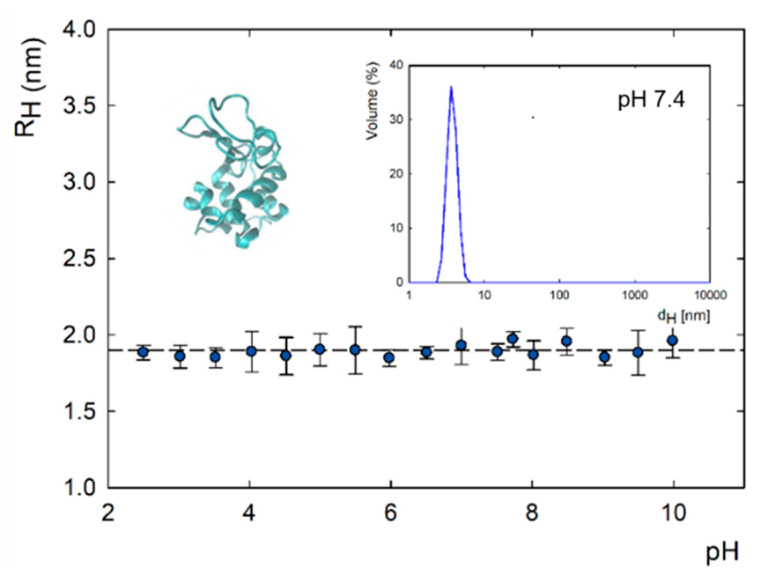
Hydrodynamic radius as a function of pH and size distribution of lysozymes in 2.5 mM TRIS-HCl in 0.05 M NaCl.

**Figure 2:**
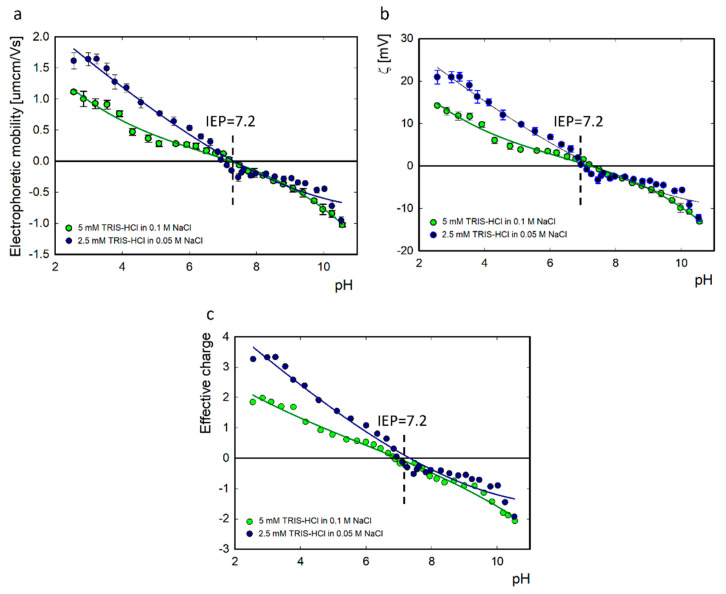
Electrophoretic mobility (**a**), zeta potential (**b**), and effective charge (**c**) of lysozymes as a function of pH at 5 mM TRIS-HCl in 0.1 M NaCl and 2.5 mM TRIS-HCl in 0.05 M NaCl.

The lysozyme molecule has an elliptical shape with dimensions of 4.5 × 3.0 × 3.0 nm^3^ [39,40,41,42,43]. The 129-amino-acid polypeptide chain contains four disulfide bridges. The molecule is composed of an α domain consisting of four short α-helices (4–15, 24–36, 89–99, and 108–115) and a β domain composed of three antiparallel β-structures (41–60), a long loop (61–78), and a short 310 helix (80–84) [44]. The size of lysozymes diluted in 2.5 mM TRIS-HCl in 0.05 M NaCl solvent in a 1:2 ratio in a concentration of 500 ppm was determined using the DLS method. The mean radius of the lysozymes was 1.9 ± 0.63 nm, and there were no changes in the pH range of 2.0–11.0 (Figure 1). The measured size was similar to previous results for lysozymes diluted in NaCl (I = 10^−2^ M). Under these conditions, the hydrodynamic diameter of the lysozymes was approximately 4 nm, and the pH ranged from 4 to 10 [45]. The measured electrophoretic mobility, zeta potential, and effective charge of lysozymes in 5 mM TRIS-HCl diluted in 0.1 M NaCl and 2.5 mM TRIS-HCl in 0.05 M NaCl solvent are summarized in Figure 2. The determined isoelectric point for lysozymes was equal to 7.2 for both ionic strengths. Below pH 7.2, lysozymes are positively charged; above this value, they have a negative charge. The isoelectric point for lysozymes in TRIS solution is shifted towards a lower pH value in the presence of NaCl. In NaCl, the isoelectric point for this protein is pH 10 [43]. Specific adsorption of solvent ions causes a change in the effective charge of the protein. The effective charge of the protein has a key influence on the molecule’s behavior at the interface [46].

### 2.2. Stability of Lysozymes in a Bulk Solution

The structural stability of lysozymes in 5 mM TRIS-HCl in 0.05 M NaCl solvent at two pH values (4.0 vs. 7.4) and a temperature range of 20–90 °C was controlled using the circular dichroism method in a UV–Vis wavelength spectral range (200–260 nm).

**Figure 3:**
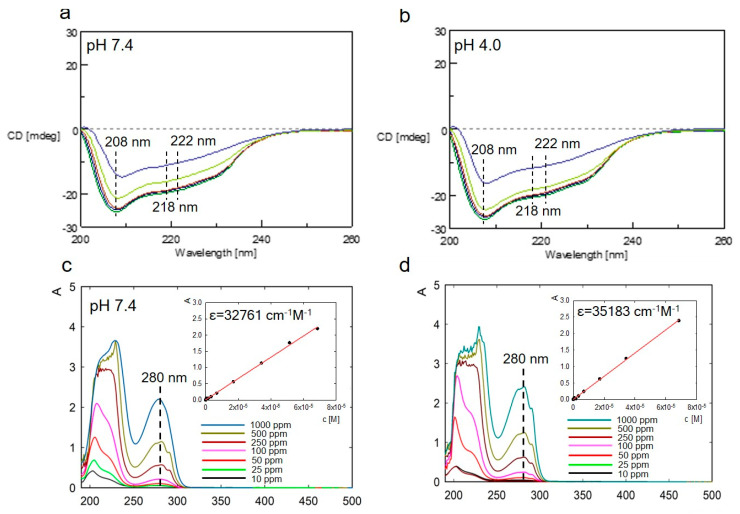
CD spectra of lysozyme in 2.5 mM TRIS-HCl in 0.05 M NaCl at pH 7.4 (**a**) and 4.0 (**b**) at different temperatures (20–90 °C). Absorption spectra of lysozymes in 2.5 mM TRIS-HCl in 0.05 M NaCl at pH 7.4 (**c**) and 4.0 (**d**) depend on the protein concentration.

In CD spectra, we observed negative bands at 208 nm and 222 nm, corresponding to the α-helical structure, and at 218 nm, characteristic of a well-defined β-sheet structure (Figure 3a,b). The obtained spectra reveal no significant structural changes at either pH condition, indicating that the native structure of lysozymes remains stable under these experimental conditions.

Literature data indicate that changes in lysozyme conformation depend on environmental conditions. In extreme environmental conditions, lysozymes are considered amyloidogenic molecules [47]. The secondary structure of lysozymes was determined based on deconvolution of the CD spectrum. The secondary structure consists of α-helices (30.1%), β-sheets (16.3%), β-turns (17.9%), and disordered structures (30.6%) at pH 4.0 and α-helices (31.2%), β-sheets (17.3%), β-turns (19.4%), and disordered structures (32.1%) at pH 7.4. The original CD spectra and models fit to them are presented in the Supplementary Materials (Figure S1).

Additionally, to confirm the conformational stability of lysozymes, UV–Vis spectra were measured in the 190–500 nm wavelength range for both pH values and at concentrations ranging from 5 to 1000 ppm (Figure 3c,d). The lysozymes yielded two absorption peaks. The first characteristic peak at around 220 nm reflects the framework conformation of the protein. The other peak at 280 nm corresponds to aromatic residues, mainly Tyr and Trp. The value of the molar absorption coefficient for this absorbance maximum (280 nm) was determined to be slightly higher for lysozymes diluted in 2.5 mM TRIS-HCl in 0.05 M NaCl at pH 4.0 than at pH 7.4 (35,183 vs. 32,761 cm^−1^ M^−1^, respectively), in line with the results obtained by the other groups [48].

### 2.3. Lysozyme Adsorption on the Gold Surface: QCM-D/MP-SPR Measurements

The lysozyme adsorption on the gold surface was monitored in parallel using the QCM-D and MP-SPR methods. The measurements were performed in a 2.5 mM TRIS-HCl solution with 0.05 M NaCl at protein concentrations ranging from 5 to 1000 ppm at pH levels of 7.4 and 4.0. The zeta potential of the gold surface at these pH conditions is equal to −30 mV at pH 7.4 and −5 mV at pH 4.0 [49]. In turn, the charge of the lysozymes under these conditions is approximately −3 mV and 18 mV, respectively. In both cases, electrostatic interactions are the primary driving forces involved in lysozyme adsorption on the gold surface. The charge distribution on the lysozyme surface is asymmetrical and depends on the pH value. Selected domains on the lysozyme surface are positively charged even if the protein’s net charge is negative. This enables the protein to interact electrostatically with the opposing surface.

**Figure 4:**
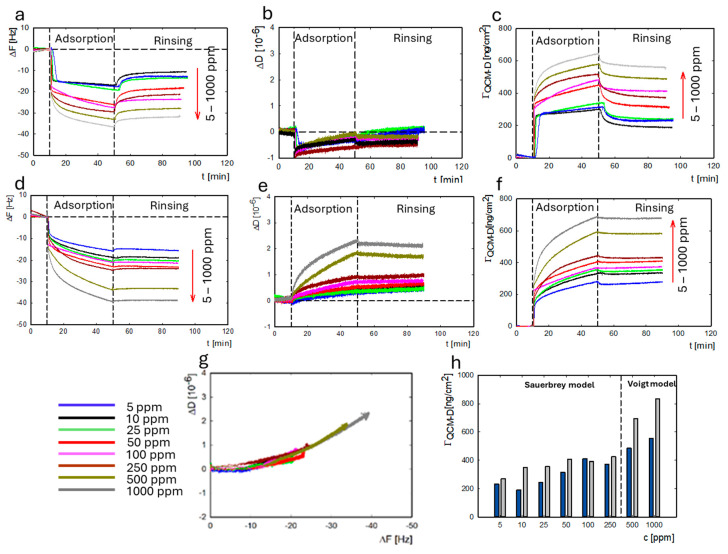
Dependence of the resonance frequency (ΔF), the dissipation ΔD, and the change in mass Γ_QCM-D_ on time for LYS adsorption at pH 7.4 (**a**–**c**) and at pH 4.0 (**d**–**f**) on the gold surface. ΔD is a function of ΔF of the QCM-D sensor as a result of LYS adsorption at pH 4.0 (**g**). The LYS mass adsorbed (Γ_QCM-D_) on the gold surface depends on the concentration at pH 7.4 (blue) and pH 4.0 (gray) (**h**).

Figure 4a–f present changes in the sensor’s resonance frequency (ΔF), dissipation (ΔD), and protein’s mass adsorbed on the gold surface as a function of time under different pH conditions. The resonance frequency data are presented from the seventh harmonic. As the protein concentration increases, the change in resonance frequency increases. The direction of change is the same for both pH values, but for pH = 4.0, the changes are larger. The changes in dissipation for pH = 7.4 are ΔD < 1 for all lysozyme concentrations. For adsorption at pH 4.0, the dissipation value was ΔD < 1 in the lysozyme concentration range of 5–250 ppm and increased to ΔD > 1.5 in the concentration range of 500–1000 ppm. The mass was calculated based on Sauerbrey’s or Voigt’s (ΔD > 1) model depending on the dissipation level (Figure 4b,e). Figure 4g,h show the change in the viscoelasticity of the lysozyme layer adsorbed on the gold surface. The changes in lysozyme mass on the gold surface at both pH values are summarized in Figure 4h. We observed an increase in mass adsorbed on the gold surface with the increase in the protein concentration. More effective lysozyme adsorption on the gold was obtained at pH 4.0. It is worth emphasizing that the mass value obtained using the QCM-D method also includes the water associated with adsorbed protein molecules.

The interaction between lysozymes and the gold surface is primarily driven by electrostatic attraction, where the dipole moment determines the direction of protein orientation. Molecular dynamics simulations showed that lysozymes adsorb to the negatively charged surface using their largest positively charged region, located on the N- and C-terminal faces. This region contains residues that play a significant role in this interaction: Lys1, Lys13, Lys95, Lys97, Arg14, and Arg128 [50].

**Figure 5:**
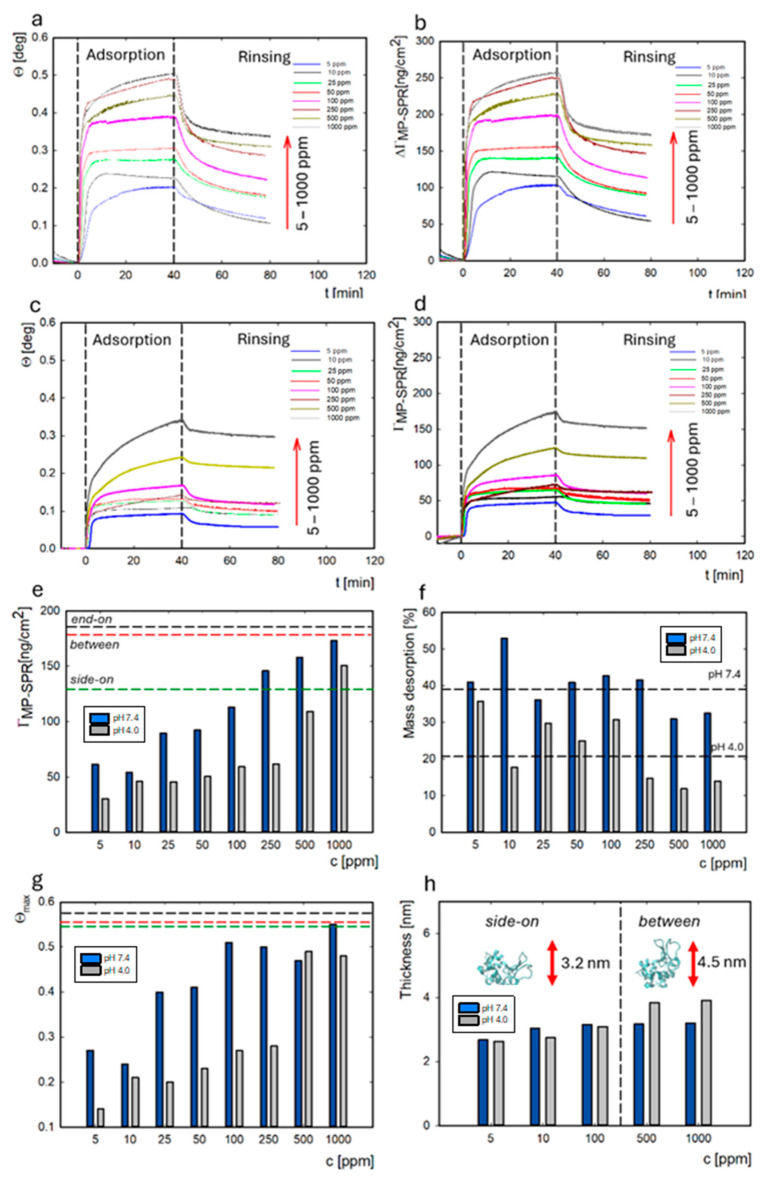
Dependence of the resonance angle and the mass Γ_MP-SPR_ on time for lysozyme adsorption at pH 7.4 (**a**,**b**) and at pH 4.0 (**c**,**d**). Adsorbed lysozyme mass on the gold surface at pH 7.4 (blue) and pH 4.0 (gray) and dashed lines corresponding to the orientations of the molecules (**e**). The percent of lysozyme mass desorbed from the gold surface at pH 7.4 vs. 4.0 depends on the protein concentration (**f**). The surface coverage (θ_max_) for the adsorbed lysozymes on the gold surface under different pH conditions. Dashed lines indicate protein orientations: side-on (green), between (red), and end-on (black) (**g**). The thickness of the lysozyme layer on the gold surface under different pH conditions (**h**).

MP-SPR measurements were performed for the same systems to determine the value of the dry mass of lysozymes on the gold surface and the thickness of the adsorbed layer. The changes in resonance angle over time for the bulk protein concentration in the range of 5–1000 ppm and changes in the mass adsorbed on the surface are presented in Figure 5a–d. The adsorption kinetics are similar to those in the QCM-D method. We observed rapid adsorption of the protein to the surface in the first stage, followed by a slow increase in adsorption in the second stage. The first stage is limited by the rate of biomolecule influx to the surface. The mass of adsorbed protein has a linear relationship with time. In the second stage, the layer gradually becomes saturated with adsorbed molecules, a process that occurs at a slower rate. As the surface layer fills, it becomes increasingly difficult to find a free adsorption site, and the molecules gradually reorient.

From the QCM-D/MP-SPR results, we found that lysozyme adsorption was more efficient at pH 4.0. The average desorption of protein mass from the surface was higher at pH 7.4 (39%) than at pH 4.0 (22%) (Figure 5f). These results were also supported by the association (k_a_) and dissociation (k_d_) rate constants calculated using the TraceDrawer software version 1.4 (Table 1). The OneToTwo kinetic model, which takes into account rearrangements and conformational changes, was selected for the presented two-step adsorption process. The overall rate constants were calculated based on two equations: k_a_ = k_a1_·k_a2_ and k_d_ = k_d1_·k_d2_. The average equilibrium dissociation constant (KD_av_) was calculated based on the values of K_D1_ and K_D2_ according to the formula below, where K_D1_ and K_D2_ are equilibrium dissociation constants for two different types of bonds and R_max1_ and R_max2_ are maximum binding capacities for sites 1 and 2.

(1) $$\mathrm{KDav}=\left(\frac{R_{\max1}\cdot K_{D1}+R_{\max2}\cdot K_{D2}}{R_{\max1}+R_{\max2}}\right)$$

The association rate constant for MP-SPR measurements was one order of magnitude higher at pH 4.0 (1.8 × 10^7^ 1/Ms) than at pH 7.4 (5.9 × 10^6^ 1/Ms). The dissociation rate constant was one order of magnitude lower (3 × 10^−9^ 1/s) at pH 4.0. The calculated average equilibrium dissociation constant (KD_av_) value was higher for adsorption at pH 4.0 for both measurement methods. In our previous work on lysozymes in 0.15 M NaCl at pH 7.5, the value of the association rate constant was one order of magnitude higher (1.45 × 10^7^ 1/Ms). The value of the dissociation constant was five orders of magnitude lower (1.16 × 10^−14^ 1/s) than in our system for data obtained from MP-SPR measurements [51].

**Scheme 1:**
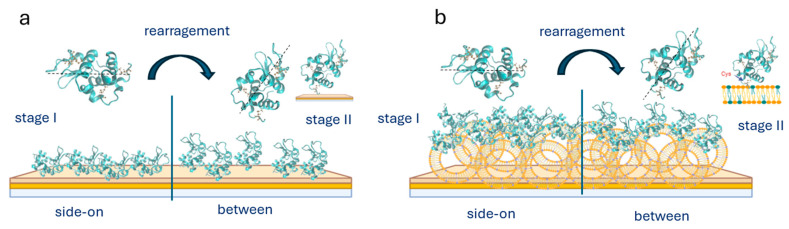
Lysozyme adsorption on the gold surface (**a**) and the liposome (**b**), indicating rearrangement of the lysozyme molecule from stage I to stage II.

It has been previously demonstrated that, during the adsorption process, as the protein concentration increases, lysozymes change their orientation from a side-on to an end-on configuration (Scheme 1) [51]. The monolayer in the side-on orientation corresponds to an adsorbed dry mass of 129.38 ng/cm^2^ according to calculations based on the random sequential adsorption model for ellipsoid-shaped molecules. In comparison, the mass is 176.91 ng/cm^2^ for the between orientation and 183.71 ng/cm^2^ for the end-on orientation. The obtained values of dry mass for adsorbed protein on the surface (MP-SPR) indicate that lysozymes have a side-on orientation in the concentration range of 5 to 100 ppm (61–113 ng/cm^2^) at pH 7.4 and from 5 to 500 ppm at pH 4.0 (30–109 ng/cm^2^). Above these concentrations, a monolayer forms and orientation changes occur between them (Figure 5e). The maximum dry mass obtained for adsorption at pH 7.4 and 4.0 was 173 ng/cm^2^ and 150 ng/cm^2^, respectively. The changes in the molecule’s orientation in the layer are related to the change in the surface coverage (θ). Based on the RSA model, the surface coverage parameters for various orientations of the lysozyme monolayer are equal to 0.577 (side-on), 0.558 (between), and 0.547 (end-on). The maximum surface coverage for the lysozyme concentration of 5–1000 ppm was calculated (Figure 5g), according to these values and the obtained dry mass. For adsorption at pH 7.4, the surface coverage (θ_max_) increases from 0.27 to 0.55 as the lysozyme concentration ranges from 5 to 1000 ppm. The same trend was observed for adsorption at pH 4.0, with an increasing surface coverage (0.14–0.48) as the protein concentration increased. These results were supported by an increase in the thickness of the adsorbed lysozyme layer to 3.9 nm at a concentration of 500–1000 ppm at pH 4.0, which confirmed a rearrangement of the molecule to a between orientation (Figure 5h).

**Figure 6:**
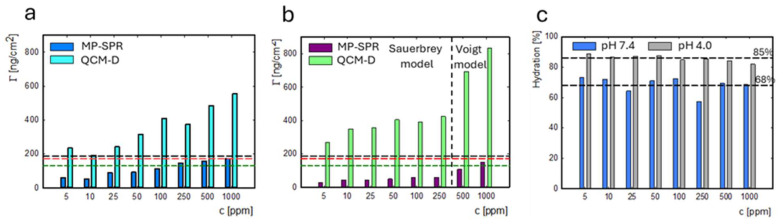
The change in mass on the gold sensor measured with QCM-D monitoring (light blue) and MP-SPR (navy) at pH 7.4 (**a**). The change in mass on the gold sensor was measured with QCM-D monitoring (green) and MP-SPR (purple) at pH 4.0 (**b**). The Voigt model was used to calculate lysozyme mass in the concentration range of 500–1000 ppm for adsorption at pH 4.0. Dashed lines indicate the mass of protein monolayer corresponding to the particular orientations of the molecules: side-on (green), between (red), and end-on (black). The percentage content of water in the adsorbed lysozyme layer under adsorption at pH 7.4 (blue) vs. pH 4.0 (gray) (**c**).

A comparison of the adsorbed masses Γ_QCM-D_ and Γ_MP-SPR_ (Figure 6a,b) allowed for determination of the hydration level of the adsorbed layer. The comparison of the degree of hydration of the adsorbed layer for the lysozyme concentration range of 5–1000 ppm at two pH values is presented in Figure 6c. The average hydration degree was equal to 85% for adsorption at pH 4.0 and was higher than at pH 7.4 (68%).

In Komorek et al.’s work [51], the lysozyme hydration level in 0.01 M NaCl at 7.5 hydration for the monolayer was 62%, and, for the bilayer, it increased to 85%. In our system (lysozymes in 5 mM TRIS-HCl, 0.05 M NaCl), the hydration level remained stable and did not change significantly with an increase in the protein concentration. Ouberai et al. [52] showed that protein solvation decreases with the increase in the protein concentration during protein adsorption on the hydrophobic surface. In contrast to previous results [41], we did not observe the formation of a protein bilayer at higher lysozyme concentrations.

### 2.4. Physicochemical Characteristics of Liposomes

**Figure 7:**
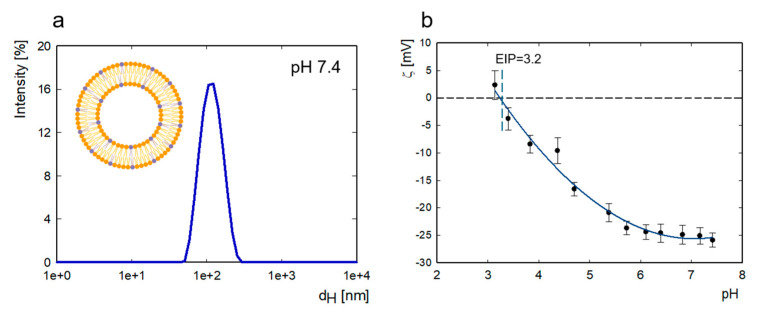
The size distribution of POPC/POPS liposomes in 5 mM TRIS-HCl, 0.15 M NaCl, and 2 mM EDTA, pH = 7.4, as measured with DLS (**a**) and the change in zeta potential of liposomes as a function of pH (**b**).

The size distribution of liposomes diluted in 5 mM TRIS-HCl and 0.15 M NaCl with 2 mM EDTA at a concentration of 1000 ppm is presented in Figure 7. Since liposomes do not contain cholesterol, we used EDTA to stabilize the liposome systems, which also has the additional effect of preventing bacterial contamination.

At both pH values, the average hydrodynamic diameter, as determined according to intensity, was 124 ± 44 nm (by volume, 103 ± 45 nm). The polydispersity index was equal to 0.1. The zeta potential of liposomes (c = 500 ppm) at pH 7.4 was −25.9 ± 1.3 mV, and the zeta potential value varied from 2.3 to −26.0 at pH levels ranging from 3.1 to 7.4. The isoelectric point for the system is located at pH = 3.2.

The POPS headgroup consists of a negatively charged phosphate group (pKa = 2.6) esterified with serine, featuring a dissociated carboxyl group (pKa = 5.5, negative charge) and an ammonium group (pKa = 11.55, positive charge) under physiological conditions. The isoelectric point of POPS in NaCl is located at pH = 4.0 [53,54]. The POPC headgroup, on the other hand, is relatively large and zwitterionic (containing a negatively charged phosphate group esterified with choline and a positively charged quaternary ammonium group; pKa = 1.0). Protonation is a pH-dependent phenomenon that affects the charge distribution and interactions of POPS lipids with other membrane components. As the degree of protonation of the carboxyl group changes, we observe a gradual decrease in the negative charge of liposomes. The contribution from the POPC molecule to the total charge of liposomes is marginal; at pH = 7.4, the POPC molecule is neutral.

### 2.5. Adsorption of Lysozymes on the Lipid Membrane Monitored with QCM-D Method

**Figure 8:**
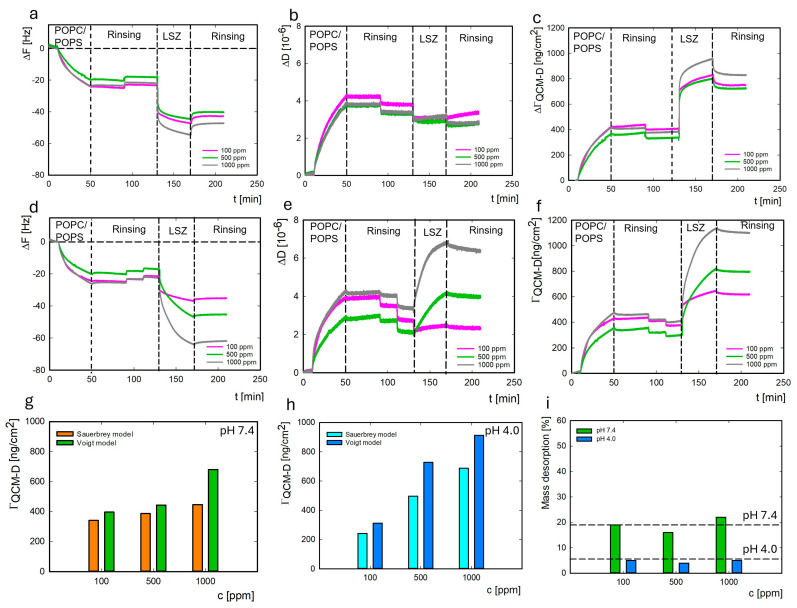
Dependence of the resonance frequency (ΔF), dissipation ΔD, and change in mass Γ_QCM-D_ on time for LYS adsorption at pH 7.4 (**a**–**c**) and at pH 4.0 (**d**–**f**) on the liposome surface. The LYS mass adsorbed (Γ_QCM-D_) on the liposome surface depends on the concentration at pH 7.4 (**g**) and pH 4.0 (**h**). The percent of lysozyme mass desorbed from the liposome surface at pH 7.4 (green) vs. 4.0 (blue) depends on the protein concentration (**i**).

To investigate the conformational stability of lysozymes on the lipid membrane, we measured lysozyme adsorption in a concentration range of 100 to 1000 ppm at pH 7.4 and 4.0 on POPC/POPS liposomes adsorbed to a gold surface. The POPC/POPS liposome sample (100 ppm) was previously diluted in 5 mM TRIS-HCl buffer (pH 7.4) containing 0.15 M NaCl. Liposomes were adsorbed for 40 min, and, after this time, the liposome layer was rinsed in two steps. For lysozyme adsorption at pH 7.4, the liposome layer was rinsed with 5 mM TRIS-HCl in 0.15 M NaCl at pH 7.4 (40 min) followed by 2.5 mM TRIS-HCl in 0.05 M NaCl at pH 7.4 (40 min). For lysozyme adsorption at pH 4.0, the liposome layer was first rinsed with 5 mM TRIS-HCl in 0.15 M NaCl at pH 7.4 (40 min), followed by 2.5 mM TRIS-HCl in 0.05 M NaCl at pH 7.4 (20 min), and finally with 2.5 mM TRIS-HCl in 0.05 M NaCl at pH 4.0 (20 min). The adsorption process was monitored using the QCM-D method, and the resulting data are presented in Figure 8a–f. In the case of adsorption on the liposome layer, the dissipation in the system exceeded 2, indicating the viscoelastic properties of the adsorbed layer. Similarly to adsorption on gold, we did not observe a change in dissipation during the adsorption of lysozymes on the liposome surface at pH 7.4. During adsorption at pH 4.0, dissipation significantly increased for lysozyme concentrations of 500–1000 ppm. A similar tendency was observed for lysozymes adsorbed on the gold surface at pH 4.0.

A review by Jackman & Cho [55] summarized the factors that influence the interaction of liposomes with an adsorption surface. Under most measurement conditions, liposome systems remain stable on gold surfaces without undergoing fusion. The vesicle fusion process depends on many factors, including the surface structure, surface charge density, and surface hydrophobicity. The absence of vesicle fusion is beneficial for verifying the interaction of biomolecules with liposome systems. Liposomes are among the most commonly used drug delivery systems. Controlling the interaction of liposome systems with proteins is an essential aspect of their application. Understanding the mechanism of interaction between liposomes and a gold surface is also crucial in the context of using gold nanoparticles in nanomedicine.

The average mass of the adsorbed liposome layer, calculated from Sauerbrey’s model, was 368 ± 43 ng/cm^2^. In comparison, the Voigt model yielded a value of 526 ± 66 ng/cm^2^. The average thickness of the adsorbed liposome layer was 5.2 ± 0.7 nm. As shown previously, the thickness of the formed lipid layer is approximately 4–6 nm, which depends on the lipid composition [56]. Due to the high dissipation value for the entire system (Figure 8b,e), the change in the mass of lysozymes adsorbed on the liposomes was also calculated using the Voigt model (Figure 8g,h). Mass calculations were performed using the QSense DFind (Biolin Scientific) software version 1.2.7, based on 3–11 frequency overtone measurements.

The established parameters were a solvent density of 1003 kg/m^3^, a protein layer density of 1113 kg/m^3^, and a POPC/POPS density of 1030 kg/m^3^. Higher efficiency of lysozyme adsorption was observed at pH 4.0. At this pH, the liposome’s surface has a charge of around −10 mV, and the charge of the protein is equal to 18 mV. As in the case of adsorption on the gold surface, higher desorption of the protein was observed at pH 7.4 (19%) than at pH 4.0 (5%) (Figure 8i). Simultaneously, the desorption of lysozymes from the liposome surface was two times lower than that from the gold surface.

The affinity of lysozyme molecules to the liposome surface was lower than to the gold at both pH conditions, based on the values of the average equilibrium dissociation constant (KD_av_) calculated using the TraceDrawer software (Table 1). Comparing the mass of protein adsorbed on the gold and liposome surfaces, we may conclude that the efficiency of lysozyme adsorption was similar for both systems (Figure S4).

### 2.6. Determination of the Conformational Stability of Lysozymes Adsorbed on the Gold Sensor and POPC/POPS Liposomes

**Figure 9:**
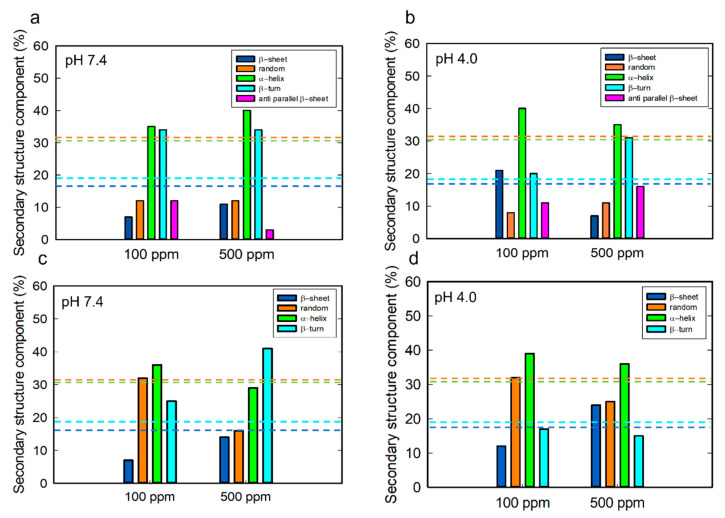
The percentage of secondary structure components of lysozymes adsorbed on the gold sensor (**a**,**b**) and liposome surface (**c**,**d**) depending on pH and the protein concentration. The dashed lines indicate the content of the secondary structure obtained from the CD spectra: navy (β-sheet), orange (random), green (α-helix), turquoise (β-turn).

FTIR spectra of the protein layer adsorbed on the gold sensor/liposome surface were recorded to determine the structural stability of lysozymes on the surface (Figure S2). The structural stability of lysozymes on the surface was investigated via deconvolution of the Amide I band, which is the most sensitive region for protein conformational changes. Figure S3 shows the deconvoluted spectral region of Amide I for a lysozyme concentration of 100 ppm. The positions of the bands characteristic for the individual components of the secondary structure were determined based on the location of maxima/minima of the II-derivative of the spectra in the range of the Amide I, which were respectively assigned as follows: 1625 cm^−1^, 1690 cm^−1^—β-sheet, 1640 cm^−1^—random coil, 1659 cm^−1^—α-helix, 1677 cm^−1^—β-turn, and 1698 cm^−1^—anti-parallel β-sheet [57,58,59,60]. The secondary structure composition of lysozymes adsorbed on both surfaces was compared. Figure 9a,b illustrate the variations in the content of lysozyme secondary structure components as a function of pH and the protein concentration for lysozymes adsorbed on the gold surface.

It has been previously demonstrated that the secondary structure composition of lysozymes changes upon adsorption to a solid surface, with the total content of disorder and β-turn structures being the dominant features—a finding also reported in our previous study [51]. Komorek et al. [43] showed that the secondary structure of lysozymes (100 ppm) adsorbed on the gold surface at pH ranging from 4.0 to 9.0 comprises 32–36% α-helices, 20–24% disorder, 15% β-sheets, and 16–18% β-turn structures at the neutral net potential.

In our study, upon adsorption on the gold surface, we observed a higher overall content of β-sheet structures (β-sheets + anti-parallel β-sheets) at both pH conditions compared to the native protein structure, as well as a decrease in disorder structure content at both pH values. Deconvolution analyses for lysozyme adsorption on the gold showed the presence of an antiparallel β-sheet structure in lysozymes.

In the case of lysozyme adsorption on the liposome surface, we found a higher overall percentage of random coil structure at both pH values compared to adsorption on the gold. We observed a decrease in disorder and α-helix structures and an increase in β-sheet content with the increase in the protein concentration at both pH values. We found a reduction in β-turn structures at pH 4.0. Changes in the composition of secondary structures with the increase in lysozyme concentration may indicate the reorientation of the protein on the surface. At a lysozyme concentration of 500 ppm, we observed the same total content of β-sheet structures (β-sheets + anti-parallel β-sheets) for lysozymes adsorbed onto both the gold and liposome surfaces.

Studies of lysozyme aggregation on a negatively charged lipid monolayer of 1,2-dipalmitoyl-sn-glycero-3-(phospho-rac-1-glycerol) (DPPG) indicate that, at pH 6, lysozymes exist in a monomeric form with a predominance of α-helices and in an oligomeric form. At pH ≤ 3, lysozymes spontaneously assemble into oligomers characterized by an antiparallel β-pleated sheet structure [23].

The strong influence of phospholipid molecules on lysozyme structure changes is confirmed by studies using sum frequency generation spectroscopy [24]. The interactions of liposomes on the lipid membrane surface include: (i) adsorption on the membrane surface by electrostatic and hydrophobic interactions, (ii) protein internalization into the hydrophobic core of the lipid bilayer, (iii) protein conformational change, and (iv) lipid phase reorganization [61].

### 2.7. X-Ray Photoelectron Spectroscopy

XPS measurements were performed in the binding energy (BE) regions of C 1s, N 1s, O 1s, and S 2p to investigate the interaction of the protein with two different types of surfaces and to support the FT-IR spectra deconvolution data (Figure S3).

**Figure 10:**
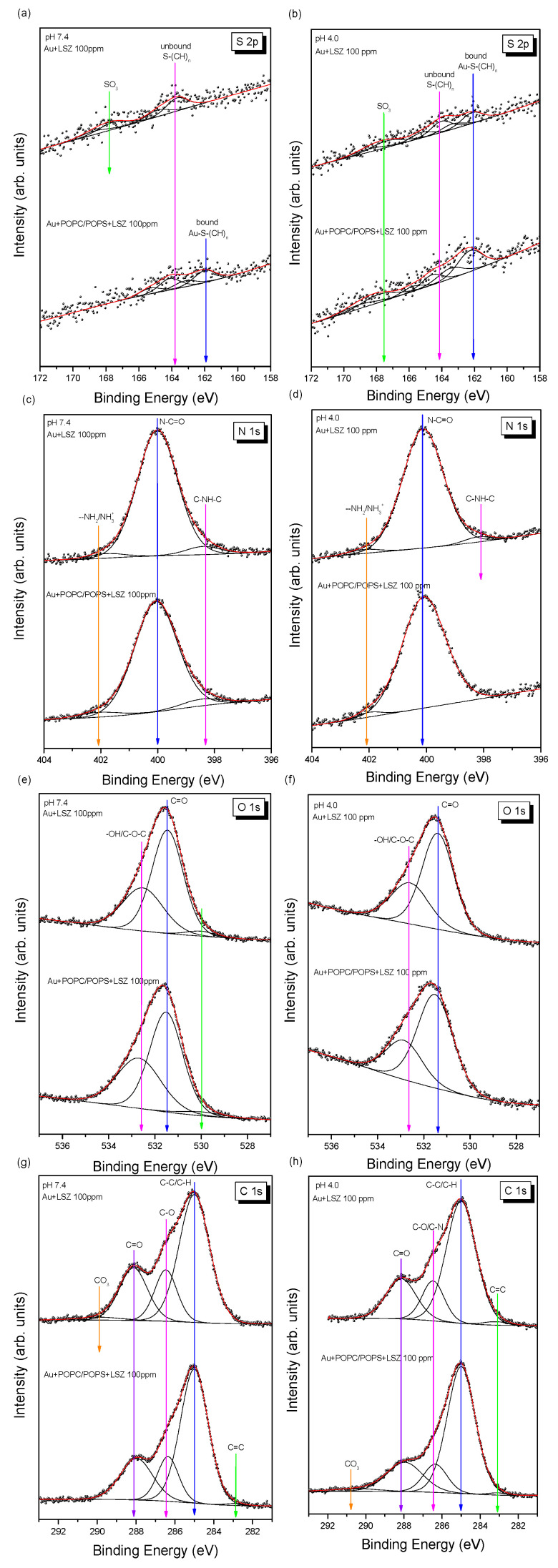
The XPS spectra of lysozymes (100 ppm) adsorbed on the gold/POPC POPS surfaces in the BE regions of S 2p at pH 7.4 (**a**), S 2p at pH 4.0 (**b**), N 1s at pH 7.4 (**c**), N 1s at pH 4.0 (**d**), O 1s at pH 7.4 (**e**), O 1s at pH 4.0 (**f**), C 1s at pH 7.4 (**g**), and C 1s at pH 4.0 (**h**).

The experimental S 2p photoemission spectra of lysozymes adsorbed on the surface of gold/liposomes at different pH values are presented in Figure 10a,b. Regarding the samples prepared at pH 4.0, three contributions with S 2p_3/2_ binding energies of 162.1, 164.1, and 167.6 eV were detected in the XPS spectra (Table 2). The component with the highest binding energy originates from SO_3_ sulfonic groups and can be attributed to native features of lysozymes [62]. The remaining components originate from sulfide groups bound to the Au substrate (162.1 eV) and unbound to the substrate (164.1 eV), as previously demonstrated in a study of thiol and disulfide interactions with gold surfaces conducted by Castner et al. [63]. These two components can be attributed to sulfur-containing amino acids, namely, cysteine residues.

The native structure of lysozymes is stabilized by four disulfide bonds: two in the α-helix domain (Cys6–Cys127 and Cys30–Cys115), one in the β-sheet domain (Cys64–Cys80), and another connecting both domains (Cys76–Cys94) [58]. Disulfide bonds ensure the thermal stability and cohesion of lysozymes, resulting in the safety of the natively folded protein. Therefore, a shift in the BE position of the sulfide group component during the adsorption of lysozymes on the surface of liposomes at both pH values could suggest an interaction between cysteines and liposomes. However, this shift was not observed in the case of lysozyme adsorption on the gold surface at either pH value, suggesting that the protein does not form a chemical bond with the Au substrate in this case.

Deconvolution of the high-resolution N 1s spectra (Figure 10c,d) revealed that most nitrogen atoms occur in a deprotonated form originating from the amine group [64,65,66]. The N 1s peak located near 400 eV can be attributed to C–N bonds and indicates the attachment of the protein to the surface [67]. Two additional components, with a small contribution to the overall spectrum, are also clearly visible, with N 1s binding energies of 398.4 eV and 402.0 eV (Table 2). The first component can be attributed to an amine-like N 1s environment [68]. The second component is associated with hydrogen-bonded/protonated amine species (–NH_2_/NH^3+^) [69]. The relative contribution of these bonds is slightly higher in samples prepared at pH 7.4.

The O 1s oxygen spectra (Figure 10e,f) are characterized by two dominant components corresponding to carbonyl (531.4 eV) and hydroxyl/ether (532.6 eV) bonds [70]. Their relative ratio is almost constant and amounts to approximately 1.9 (Table 2). Carbonyl groups can be attributed to the peptidic oxygen of protein (O–C=O and N–C=O) [51,58]. In samples prepared at pH 7.4, a minor component (~3%) with a binding energy of 530.0 eV is present, the origin of which is unknown.

The C 1s carbon lines (Figure 10g,h) are slightly more complex and can be well fitted using five components: (i) C–C/C–H bonds, (ii) C–O/C–N groups, (iii) C=O carbonyl groups, (iv) CO_3_ carbonate groups, and (v) C=C double bonds. In addition to the dominant component (60%) originating from aliphatic C–C/C–H bonds, two other functional groups (C–O/C–N and C=O/N–C=O) contribute significantly to the total spectrum, which is consistent with recent publications [68,71]. The shape of the C 1s spectrum is very similar to that of mesocellular silica foam coated by lysozymes [65] and lysozymes on stainless steel [72]. The carbonate and C=C components are small and do not exceed 3% of the total spectrum area (Table 2). It is worth mentioning that the shapes of C 1s and O 1s spectra do not change significantly with pH.

## 3. Materials and Methods

### 3.1. Materials

Lysozymes from chicken egg white purchased from Sigma Aldrich (Burlington, MO, USA) were used for the research. 1-Palmitoyl-2-oleoyl-glycero-3-phosphocholine (POPC) and 1-palmitoyl-2-oleoyl-sn-glycero-3-phospho-L-serine (POPS) were acquired from Avanti Polar Lipids (Alabaster, AL, USA). Anhydrous chloroform (cat. No. 288306) was obtained from Sigma Aldrich, TRIS base (Ultra-pure, BioShop, Burlington, ON, Canada) was procured from LabEmpire (Rzeszów, Poland), and sodium chloride was acquired from Sigma Aldrich. TRIS-HCl buffer was prepared by dissolving 5 mM of TRIS base, 0.15 M NaCl, and 0.1 M HCl (to adjust pH to 7.4). Lysozyme solutions were prepared by dissolving protein powder in 2.5 mM TRIS-HCl in 0.05 M NaCl at pH 7.4 or 4.0. QCM-D sensors with gold (QSX 301) with a fundamental frequency of 5 MHz were purchased from Biolin Scientific (Gothenburg, Sweden).

### 3.2. Liposome Preparation

POPC/POPS (4:1) liposomes were prepared using the Thin-Film Hydration (TFH) method followed by extrusion [73]. Appropriate amounts of lipids were placed in a round-bottom flask and dissolved in anhydrous chloroform to obtain a total lipid concentration of 10 mg/mL. Then, chloroform was evaporated under a nitrogen stream, and the lipid film was placed in a desiccator under reduced pressure for 48 h. After removal of the organic solvent, the dry lipid film was hydrated in 5 mM TRIS-HCl buffer containing 0.15 M NaCl and 2 mM EDTA. The lipid mixture was dispersed via vortexing and subjected to five freeze–thaw cycles in liquid nitrogen and water at 60 °C. At the final stage, the mix of multilamellar vesicles was extruded through polycarbonate membranes with pore sizes of 400 nm, 200 nm, and 100 nm using a high-pressure extruder. The mean hydrodynamic radius, polydispersity index, and electrophoretic mobility of liposomes were determined using a Malvern Nano ZS analyzer (Worcestershire, UK).

### 3.3. Methods

#### 3.3.1. Dynamic Light Scattering

The size (hydrodynamic diameter) of lysozymes and liposomes was determined using the dynamic light scattering (DLS) method with a Malvern Zetasizer Nano ZS instrument equipped with a 4 mW He–Ne laser operating at a wavelength of 633 nm and a fixed detector angle of 173°. The measured sample was diluted in 2.5 mM TRIS-HCl in 0.05 M NaCl (for proteins) or 5 mM TRIS-HCl in 0.15 M NaCl (for liposomes) to a final concentration of 1000 ppm. The hydrodynamic diameter was calculated using the Stokes–Einstein equation:

R_H_ = kT/(6πηD) (2)

where k is the Boltzmann constant; T is the absolute temperature; η is medium viscosity; and D is the diffusion coefficient.

#### 3.3.2. Electrophoretic Mobility Measurements

Electrophoretic mobility (µe) was determined using a Malvern Nano ZS analyzer (Worcestershire, UK). Measurements were conducted in the pH range of 2.0–11.0. The zeta potential value was calculated from Henry’s equation. The Smoluchowski limit f(κa) = 1.5 was applied for the calculations. The concentration of the measured sample was equal to 1000 ppm. The pH of the solutions was adjusted using sodium hydroxide (NaOH) with a concentration of 0.1 M and hydrochloric acid (HCl) with a concentration of 0.05 M.

#### 3.3.3. UV–Vis Spectrometry

A Thermo Scientific Evolution 201 UV–Vis spectrometer was applied to determine the stability of lysozymes in solution. The UV–Vis spectra were measured in the 190–500 nm wavelength range in 1 cm quartz cuvettes.

#### 3.3.4. Circular Dichroism

The structure of lysozymes in a 2.5 mM TRIS-HCl in 0.05 M NaCl solvent at varied temperatures (20–90 °C) and two pH values (4.0 vs. 7.4) was monitored with Circular Dichroism (CD) using a Jasco-1500 spectrometer with a 150 W Xe lamp. The concentration of the lysozyme solution was 100 ppm. The CD spectra were measured in the far UV–Vis range between 200 and 260 nm with a resolution of 1 nm and a scanning speed of 50 nm/min.

#### 3.3.5. Quartz Crystal Microbalance with Dissipation (QCM-D) Monitoring

The protein adsorption process on the gold surface and the lipid membrane was monitored using a quartz crystal microbalance with energy dissipation monitoring (Q-Sense E1 device). In the first step, 2.5 mM TRIS-HCl in 0.05 M NaCl at pH 7.4 or 4.0 (for proteins) or 5 mM TRIS-HCl in 0.15 M NaCl at pH 7.4 (for liposomes) buffer was injected to record the baseline. All solutions were injected at a flow rate of 0.5 mL/min and 25 °C. Depending on the dissipation value, the mass adsorbed on the surface was calculated based on Sauerbrey’s or Voigt’s model.

#### 3.3.6. Multi-Parametric Surface Plasmon Resonance (MP-SPR)

The adsorption of lysozymes on the gold surface at concentrations ranging from 5 to 1000 ppm and at two pH values (7.4 and 4.0) was simultaneously monitored using the MP-SPR method. The measurements were conducted using an SPR NaviTM 200 instrument (BioNavis). A series of measurements was performed for angles ranging from 55 to 75° for a solution flow rate of 50 μL/min at a temperature of 25 °C. The adsorbed mass was calculated based on the change in resonance angle Δθ and refractive index increment (dn/dc) from the formula

(3) $$\Gamma_{\mathrm{MP}\text{-}\mathrm{SPR}}=\frac{{\Delta \theta \mathrm{kd}}_{\mathrm{LYS}}}{\frac{\mathrm{dn}}{\mathrm{dc}}}$$

where Δθ is the change in the SPR angle, k is the SPR instrument constant, d_LYS_ is the assumed thickness of the adsorbed lysozyme layer, and dn/dc is the refractive index increment. The increment was determined by measuring the refractive index with an Atago RX-5000α refractometer (in bulk lysozyme solution). The refractive index increment was calculated based on the Perlmann and Longsworth formula. The dn/dc for lysozymes in 2.5 mM TRIS-HCl in 0.05 M NaCl was equal to 0.196 cm^3^/g for λ = 670 nm. The value of kd_LYS_ = 1.0 × 10^−7^ nm/deg was applied in the above formula. The thickness and refractive index of the layer were determined using the computer software MP-SPR NaviTM LayeSolver v. 1.4.0.3.

#### 3.3.7. Fourier Transform Infrared Spectroscopy (FTIR)

FTIR absorbance spectra were collected using a Nicolet iS50 (Thermo Fisher Scientific, Boston, MA, USA) with an accessory for the specular reflection method. The gold sensor with an adsorbed protein layer was measured in the mid-infrared range (4000–400 cm^−1^) at room temperature, and 512 scans were co-added at a nominal resolution of 4 cm^−1^. The percentage of the lysozymes’ secondary structure components was determined via deconvolution of the Amide I band in the OriginPro 2023 Software.

#### 3.3.8. X-Ray Photoelectron Spectroscopy (XPS)

X-ray photoelectron spectroscopy (XPS) studies were carried out in a multi-chamber UHV system equipped with a SES R4000 hemispheric energy analyzer (Gammadata Scienta) and a non-monochromatic MgK_α_ source (12 kV, 15 mA). Details of XPS measurements have been presented elsewhere [74]. Since the tested materials were weakly conductive and applied on a gold sensor, the whole system behaved like a capacitor in XPS studies, and all spectral lines were significantly shifted. To remove this effect, a calibration was performed on the aliphatic (C–C/C–H) component of the C 1s spectrum (set at a binding energy of 285.0 eV) and validated using the Au 4f_7/2_ line at 84.0 eV [63]. A spin–orbit splitting of 1.2 eV was used to fit the S 2p spectra numerically.

## 4. Conclusions

The interactions between proteins and solid surfaces are crucial phenomena that influence numerous biological and industrial processes. Therefore, multifaceted characterization of proteins in the adsorption process can contribute to elucidating the relationship between the structure and properties of biomolecules. The behaviors of lysozymes on gold and lipid membrane surfaces were monitored using quartz crystal microbalance with dissipation (QCM-D) monitoring, multi-parametric surface plasmon resonance (MP-SPR), infrared spectroscopy (FTIR), and XPS. Particular attention was paid to the structural stability of the protein and the hydration of protein layers formed at the interface. MP-SPR and QCM-D studies indicate that the adsorption of lysozymes on both surfaces was more efficient at pH 4.0 than 7.4. However, the average equilibrium dissociation constants (KDav.) indicate a lower affinity of lysozymes for gold and a higher affinity for the lipid membrane surface at pH 4.0. Based on the KDav constant values, the affinity of lysozyme molecules to the lipid membrane surface was lower than to the gold surface at both pH values. Regardless of the adsorption surface, when the protein is positively charged, the lysozyme layer adsorbed at pH 4.0 shows higher viscoelastic properties. The differences in the FTIR spectra—and, thus, in the composition of the secondary structures of lysozymes as a result of adsorption—confirm the different mechanisms of interaction of the protein with the adsorption surfaces. The composition of the secondary structures of lysozymes depends on the type of surface, pH conditions, and surface coverage. Amide I deconvolution revealed the presence of an antiparallel β-sheet structure during lysozyme adsorption on the gold surface, which was not observed on the lipid membrane surface. As a result of the interaction of lysozymes with the gold surface, we observed a decrease in the content of disordered structures alongside an increase in the content of β-sheet structures (parallel + antiparallel) at both pH conditions. Lysozyme adsorption on the liposome surface was found to be characterized by a higher proportion of disordered structures at both pH values, compared to gold adsorption. Moreover, there was an increase in β-sheets and a decrease in α-helix and disordered structures with an increase in surface coverage. XPS spectra revealed a shift in the band position assigned to the S-(CH)n bond as a result of lysozyme adsorption on the lipid membrane surface at both pH values, which may confirm that, in this case, the cysteine-containing domains participate in the interaction with the adsorption surface. The obtained results confirm that electrostatic interactions primarily drive the interaction of lysozymes with the negatively charged gold surface or with the negatively charged lipid membrane surface.

## Figures and Tables

**Table 1:** The overall association (k_a_) and dissociation (k_d_) rate constants were calculated based on fitting the OneToTwo kinetic model using TraceDrawer (Ridgeview Instruments AB, Uppsala, Sweden).

Method, pH	Overall Association Rate Constant (k_a_) (1/Ms)	Overall Dissociation Rate Constant (k_d_) (1/s)	KD_av_ (M)
	Lysozymes on Au		
MP-SPR, pH 7.4	5.9 × 10^6^	3.6 × 10^−8^	2.0 × 10^−7^
QCM-D, pH 7.4	1.3 × 10^8^	1.3 × 10^−13^	2.9 × 10^−8^
MP-SPR, pH 4.0	1.8 × 10^7^	3.0 × 10^−9^	7.3 × 10^−7^
QCM-D, pH 4.0	7.5 × 10^7^	6.3 × 10^−16^	1.5 × 10^−7^
	Lysozymes on POPC/POPS		
QCM-D, pH 7.4	7.9 × 10^7^	2.7 × 10^−15^	2.0 × 10^−6^
QCM-D, pH 4.0	4.6 × 10^7^	8.9 × 10^−16^	9.3 × 10^−7^

**Table 2:** The BE values (eV) and relative areas of components (%) of S 2p_3/2_, N 1s, O 1s, and C 1s peaks for all samples.

Line	Au-LSZ pH 7.4		Au-POPC/POPS pH 7.4		Au-LSZ pH 4.0		Au-POPC/POPS pH 4.0		Classification
	BE (eV)	Area (%)	BE (eV)	Area (%)	BE (eV)	Area (%)	BE (eV)	Area (%)	
S 2p_3/2_	---	---	162.0	52.7	162.1	34.3	162.3	52.3	bound Au–S–(CH)_n_
	163.7	58.3	164.1	47.3	164.0	34.6	164.6	20.6	unbound S–(CH)_n_
	167.6	41.7	---	---	167.4	31.1	167.9	27.1	SO_3_
N 1s	398.4	5.2	398.5	5.1	398.2	2.7	---	---	C–NH–C
	400.0	91.8	400.1	91.1	400.1	95.0	400.1	97.7	C–NH_2_
	401.8	3.0	402.1	3.8	402.2	2.3	402.0	2.3	–NH_2_/NH_3_^+^
O 1s	530.0	2.7	530.0	2.8	---	---	---	---	?
	531.4	63.0	531.5	58.1	531.4	66.3	531.5	69.9	C=O
	532.5	34.3	532.7	39.1	532.6	33.7	532.9	30.1	–OH/C–O–C
C 1s	---	---	282.9	0.7	283.2	1.4	283.1	0.8	C=C
	285.0	58.6	285.0	61.6	285.0	62.7	285.0	65.6	C–C/C–H
	286.5	17.2	286.4	15.5	286.5	15.2	286.4	12.3	C–O/C–N
	288.1	22.4	288.0	22.2	288.2	20.7	288.0	19.2	C=O
	289.8	1.8	---	---	---	---	290.5	2.1	CO_3_

## Data Availability

The original contributions presented in this study are included in the article/Supplementary Materials.

## Supplementary Materials

The following supporting information can be downloaded at: https://www.mdpi.com/article/10.3390/ijms262311303/s1.

## Abbreviations

The following abbreviations are used in this manuscript:

BE	Binding energy
CD	Circular dichroism
D	Diffusion coefficient
DLS	Dynamic light scattering
FTIR	Fourier Transform Infrared Spectroscopy
k_a_	Association rate constant
k_d_	Dissociation rate constant
KD_av_	Equilibrium dissociation constant
MP-SPR	Multi-parametric surface plasmon resonance
QCM-D	Quartz crystal microbalance with dissipation
POPC	1-Palmitoyl-2-oleoyl-glycero-3-phosphocholine
POPS	1-Palmitoyl-2-oleoyl-sn-glycero-3-phospho-L-serine
R_H_	Hydrodynamic radius
XPS	X-ray photoelectron spectroscopy
