# Supplementary material for: Role of the Surface in Conformational Changes in Lysozymes: Effect of a Gold Surface and a Lipid Membrane
Source: Int J Mol Sci. 2025 Nov 22;26(23):11303. doi: 10.3390/ijms262311303 (PMC12692700; doi:10.3390/ijms262311303)
Supplement: Supplementary file 1 [file ijms-26-11303-s001.zip › ijms-3999643-supplementary.pdf]

Supporting Information

Role of the Surface in Conformational Changes in Lysozymes: Effect of a Gold Surface and a Lipid Membrane.

Agnieszka Kaminska[1], Lukasz Lustyk[1], Jacek Gurgul[1], Barbara Jachimska[1*]

[1]Jerzy Haber Institute of Catalysis and Surface Chemistry, Polish Academy of Sciences, Niezapominajek 8, 30-239 Krakow, Poland

*Correspondence to Professor Barbara Jachimska, Jerzy Haber Institute of Catalysis and Surface Chemistry, Polish Academy of Sciences,
e-mail: barbara.jachimska@ikifp.edu.pl

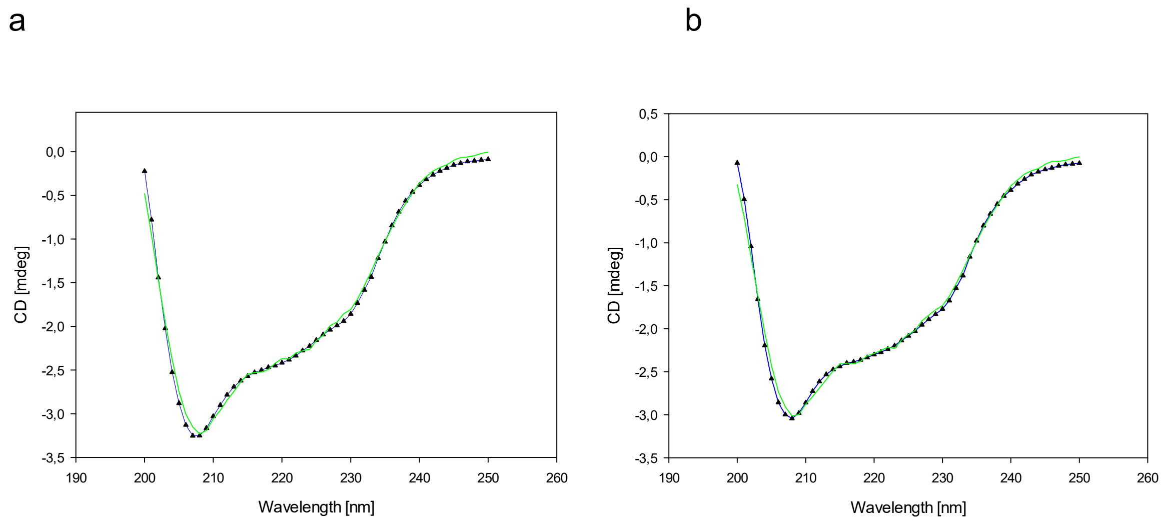

Figure S1. CD spectra of lysozyme in 2.5 mM TRIS-HCl in 0.05 M NaCl at pH 7.4 (**a**) and 4.0 (**b**). The black points and green lines denote the experimental data and the fitted curve, respectively, using the BeStSel web server.

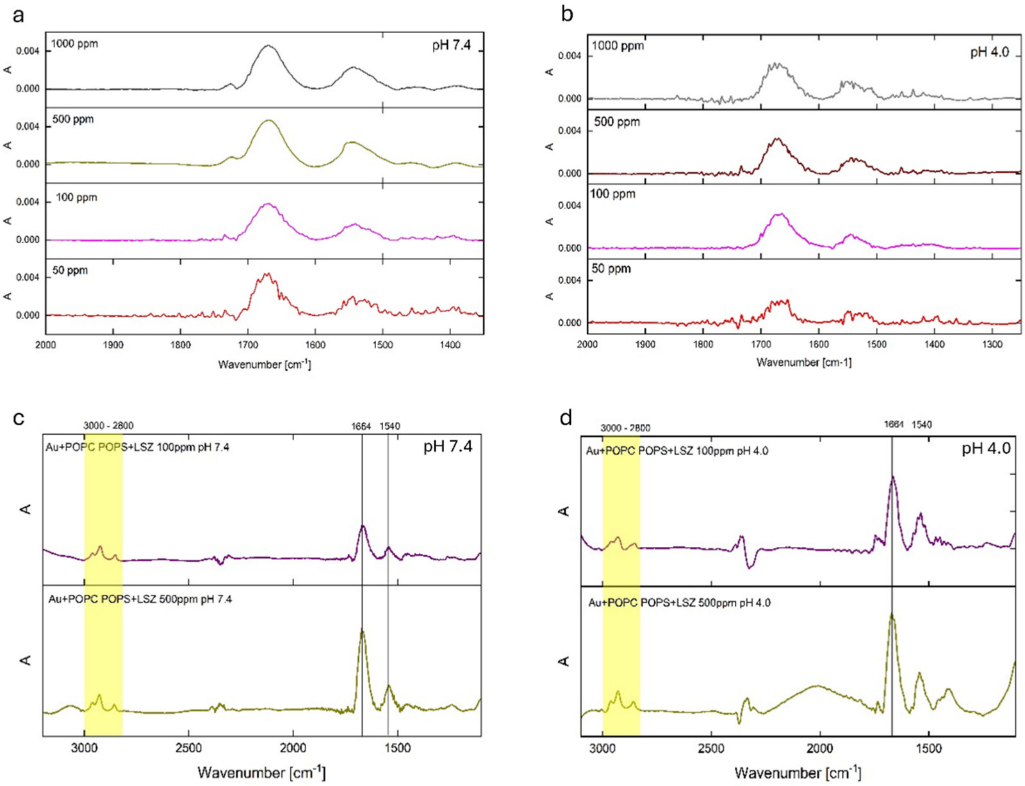

Figure S2. FT-IR spectra recorded for lysozyme adsorbed on the gold surface at pH 7.4 and 4.0 in the concentration range 50-1000 ppm (**a-b**) and on the liposome surface at pH 7.4 and 4.0 in the concentration range 100-500 ppm (**c-d**).

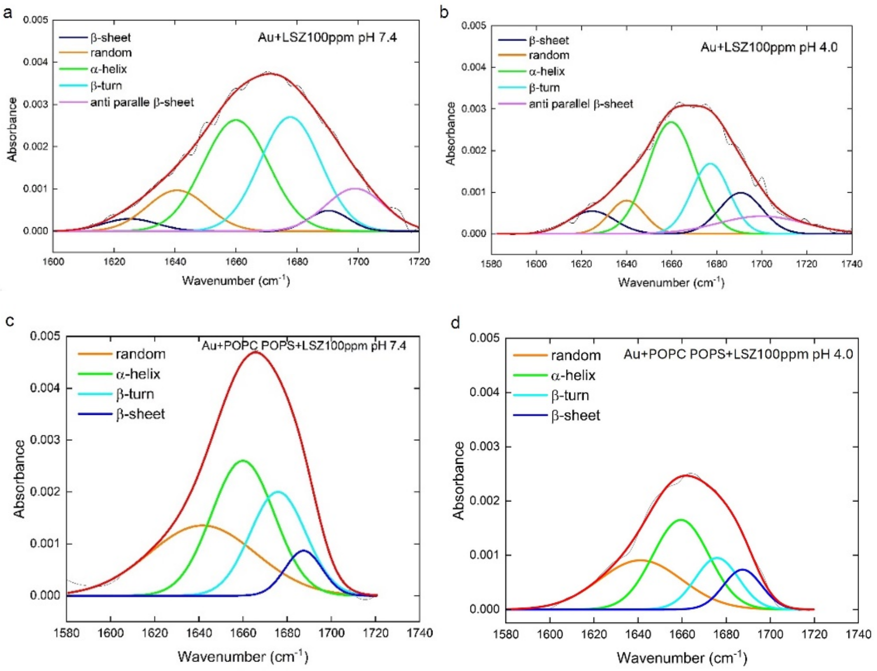

Figure S3. Deconvoluted FT-IR spectrum in the Amide I range for lysozyme (100 ppm) adsorbed on the gold (**a-b**) and POPC/POPS liposomes surface (**c-d**) at different pH conditions.

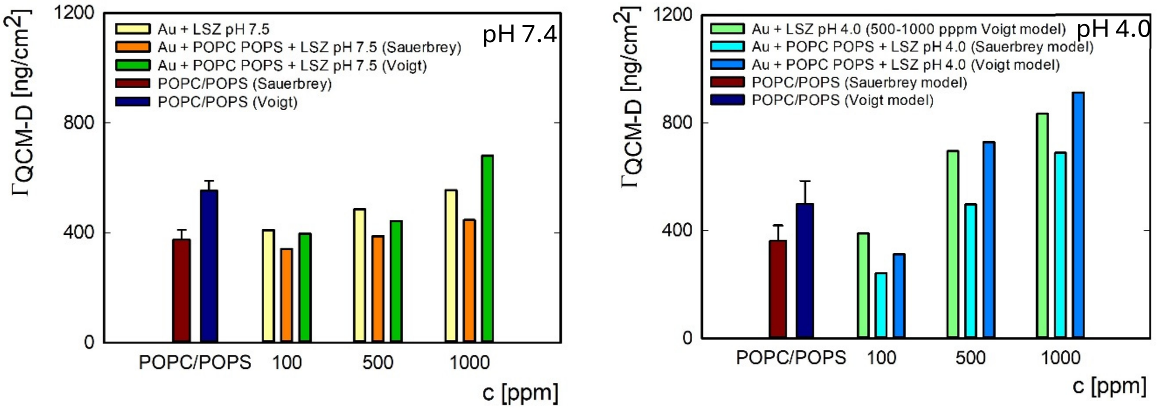

Figure S4. Comparison of the masses of lysozyme adsorbed on the gold and liposome surfaces at different pH conditions. Additionally, the mass was calculated using Sauerbrey’s and Voigt's models.
